# Assessing the role of livestock and sympatric wild ruminants in spreading antimicrobial resistant *Campylobacter* and *Salmonella* in alpine ecosystems

**DOI:** 10.1186/s12917-021-02784-2

**Published:** 2021-02-15

**Authors:** Johan Espunyes, Oscar Cabezón, Andrea Dias-Alves, Pol Miralles, Teresa Ayats, Marta Cerdà-Cuéllar

**Affiliations:** 1 Wildlife Conservation Medicine Research Group (WildCoM), Departament de Medicina i Cirurgia Animals, Universitat Autònoma de Barcelona, Bellaterra, Spain; 2 Research and Conservation Department, Zoo de Barcelona, Barcelona, Spain; 3 UAB, Centre de Recerca en Sanitat Animal (CReSA, IRTA-UAB), Campus de la Universitat Autònoma de Barcelona, Bellaterra, Barcelona, Spain; 4 IRTA, Centre de Recerca en Sanitat Animal (CReSA, IRTA-UAB), Campus de la Universitat Autònoma de Barcelona, Bellaterra, Barcelona, Spain

**Keywords:** *Campylobacter*, *Salmonella*, Antimicrobial resistance, Free-ranging livestock, Pyrenean chamois

## Abstract

**Background:**

Livestock play an important role as reservoir of enteric pathogens and antimicrobial resistance (AMR), a health and economic concern worldwide. However, little is known regarding the transmission and maintenance of these pathogens at the wildlife-livestock interface. In this study, we assessed the occurrence, genetic diversity and AMR of *Campylobacter* spp. and *Salmonella* spp. shed by sympatric free-ranging livestock and a wild herbivore in an alpine ecosystem.

**Results:**

*Campylobacter* spp. was isolated from 23.3 % of cattle and 7.7 % of sheep but was not isolated from horses nor Pyrenean chamois (*Rupicapra pyrenaica*). *Campylobacter jejuni* was the most frequent species. A high genetic diversity and certain host specificity of *C. jejuni* isolates was observed. The main AMR detected in *Campylobacter* isolates was to nalidixic acid (88.2 %), ciprofloxacin (82.4 %) and tetracycline (82.4 %); only 11.7 % of the isolates were pan-susceptible and 17.6 % were multi-resistant. *Salmonella* ser. Newport was isolated only from one Pyrenean chamois and was pan-susceptible.

**Conclusions:**

Results show that free-ranging cattle and sheep are spreaders of *Campylobacter* as well as their AMR strains in the alpine environment. Therefore, contaminated alpine pastures or streams may constitute a source for the dissemination of AMR enteropathogens. However, apparently, alpine wild ungulates such as Pyrenean chamois play a negligible role in the epidemiology of zoonotic enteropathogens and AMR, and are not potential bioindicators of the burden of alpine environments.

## Background

Gastrointestinal foodborne infections are challenging issues for humanity. They cause an estimated number of 600 million illnesses and 420,000 deaths worldwide every year [1]. *Campylobacter* spp. and *Salmonella* spp. are the leading causes of these diarrhoeal diseases [1] and are also the two most reported zoonosis in the European Union (94 % of all the reported cases of zoonotic diseases in 2018; [2]). Humans get infected by these enteric bacteria mostly through handling and consumption of contaminated foods but also through direct contact with carrier animals and contaminated environments [3, 4].

Livestock act as carriers of these enteric pathogens and they play an important role in their dissemination, particularly in farm environments [5, 6]. However, there is a lack of knowledge regarding the contribution of free-ranging livestock in the epidemiology of zoonotic bacterial enteropathogens in natural environments. Livestock is usually considered to be the primary source of freshwater bacterial contamination but their effect on the bacterial quality of soils is rarely assessed [7]. The role of wildlife in the ecology of *Campylobacter* and *Salmonella* is also poorly understood, mostly due to the unknown carrier status of many wild species. Several studies have identified wild animals as potential environmental reservoirs of food borne pathogens [8, 9]. For example, wild birds such as seagulls and waterfowl have been shown to have a role in the dispersion of these diseases [10, 11]. However, the wildlife compartment is also considered a “net sink” of enteric pathogens due to a contamination from domestic species or human waste [12, 13]. Nevertheless, little is known regarding the transmission of those pathogens at the wildlife-livestock interface. Transmission may be bidirectional [14], but at the same time some wild species are independent from this transmission cycle due to ecological and behavioural differentiations [15].

A more recent public health issue is related with the emergence and spread of antimicrobial-resistant pathogens in natural environments. This phenomenon has serious implications for human as well as animal health and it is considered one of the biggest threats to modern medicine [16]. Antimicrobial resistance (AMR) can be dispersed in the environment through several mechanisms, including water contaminated by sewage containing antimicrobials and resistant bacteria, run-off from fertilized land, or through the faeces of treated livestock [17]. In fact, the occurrence of AMR in livestock is considered extremely high, especially for tetracyclines, quinolones and sulphonamides [18]. The agricultural use of antibiotics can have a dramatic impact on human health and is one of the major driver of AMR worldwide [19, 20].

On the contrary, wildlife is not usually exposed to antimicrobials used in veterinary or human medicine. However, they may acquire resistant bacteria from contaminated environments, becoming new host reservoirs [21]. Despite the knowledge regarding the potential spill-over from free-ranging livestock to wildlife is still limited [21], some authors have detected a significant gradient of antibiotic resistance from wildlife less impacted by livestock production to wildlife in close contact with farm animals [22]. In fact, wildlife has been suggested as bioindicators or sentinels of the AMR burden present in the environment, even if the specific transmission routes still have to be elucidated [23, 24].

When considering less impacted habitats and remote regions of the world, the origin of the burden of resistance within the local environment can be narrowed down to limited inputs. In high-altitude alpine environments, where human waste disposal, wastewater runoff and sewage contamination can be considered absent, the only apparent important source of AMR is the presence of free-ranging livestock during the summer months. In mountainous areas of Europe, traditional farming practices are based on livestock freely grazing on alpine grasslands from after the snowmelt until the decay of grass quality in autumn, usually from May to October. This practice is frequently applied to reduce the economic costs involved in livestock maintenance. As such, it is easier to assess the contribution of livestock to the prevalence of AMR pathogens in wildlife, as numerous other anthropogenic inputs are absent.

In this study, we assessed the shedding of *Campylobacter* spp. and *Salmonella* spp. and their AMR strains by sympatric free-ranging livestock (cattle, sheep and horses) and a wild herbivore (Pyrenean chamois; *Rupicapra pyrenaica*) in an alpine ecosystem of the Eastern Pyrenees. We further explored the transfer of these bacteria between species by investigating the genetic relationship among isolates.

## Results

We isolated *Campylobacter* spp. from 17 cattle (23.3 %; CI_95 %_: 15.1–34.1) and 3 sheep (7.7 %; CI_95 %_: 2.6–20.3), whilst *Salmonella* was isolated from only one Pyrenean chamois (1.2 %; CI_95 %_: 0.2–6.3) (Table 1). None of the two pathogens were isolated from horses. At the herd level, 11 cattle herds (57.8 %) and 2 sheep flocks (66.6 %) presented at least one positive individual.

**Table 1:** Frequency of *Campylobacter* species and *Salmonella* serovars in faecal samples from livestock and Pyrenean chamois in the Freser-Setcases National Game Reserve, Eastern Pyrenees, Spain

	Cattle	Sheep	Horse	Chamois
*Campylobacter* spp.				
* C. jejuni*	16/74	3/39	0/30	0/86
* C. coli*	2/74	0/39	0/30	0/86
Total	17/74	3/39	0/30	0/86
*Salmonella* spp.				
* S.* ser. Newport	0/74	0/39	0/30	1/86
Total	0/74	0/39	0/30	1/86

In cattle, *Campylobacter jejuni* was the most frequently isolated species (16/17), followed by *Campylobacter coli* (2/17; Table 1). One animal was coinfected with both species. In sheep, *C. jejuni* was the only species isolated (3/3). We isolated *Salmonella* from only one Pyrenean chamois and all *Salmonella* isolates from this positive individual showed the same ERIC-PCR profile and were identified as *Salmonella* ser. Newport.

To assess the genetic relationship among *Campylobacter* isolates, 32 *C. jejuni* isolates (from twelve cattle and three sheep) and one *C. coli* isolate (from one cattle) were genotyped by flaA-RFLP (restriction fragment length polymorphism of the *fla*A gene). The remaining isolates could not be analysed since they were non-typeable and no band patterns were obtained.

**Fig. 1:**
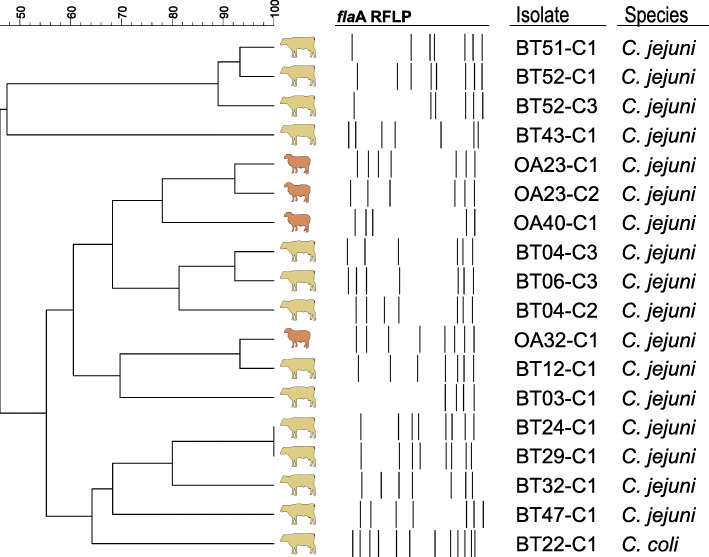
Dendrogram of *fla*A-RFLP banding patterns of *Campylobacter* spp. isolates originating from faecal samples from cattle and sheep in the Freser-Setcases National Game Reserve, Eastern Pyrenees, Spain. One different profile per animal is shown

A high genetic diversity was observed, since almost each *Campylobacter-*positive individual carried a single and unique genotype. Overall, the *fla*A-RFLP analysis revealed 13 different profiles (10 from cattle, 2 from sheep, and one shared between both host species; Fig. 1). A certain host specificity was observed, as isolates from the same host species clustered together at different similarity levels, except a *C. jejuni* isolate from cattle showing > 90 % similarity with a sheep isolate. Besides, in most cases, closely related isolates originated from individuals from the same herd.

We tested eighteen isolates from the 13 different *fla*A-RFLP profiles (Fig. 1) for antimicrobial susceptibility to a panel of six antimicrobials. One of them showed no growth in any of the wells (including the control wells) and thus no data could be obtained. MIC test was repeated three times with this strain, obtaining the same results. In overall, the main AMR detected was to nalidixic acid (88.2 %, 15/17), ciprofloxacin (82.4 %, 14/17) and tetracycline (82.4 %, 14/17), consequently the most frequent AMR profile was NalCipTet (Table 2). Two isolates, both from cattle, were pan-susceptible and three isolates, all from cattle, were multidrug resistant (MDR). The *Salmonella* ser. Newport tested was pansusceptible.

**Table 2:** Antimicrobial resistance patterns of *C. jejuni* and *C. coli* isolates originating from faecal samples from cattle and sheep in the Freser-Setcases National Game Reserve, Eastern Pyrenees, Spain

AMR profile^a^	Antimicrobial class^b^	Isolates
NAL, TET, ERY, GEN	4	1
NAL, CIP, TET, ERY	3	2
NAL, CIP, TET	2	11^c^
NAL, CIP	1	1
Pan-susceptible	-	2

^a^Quinolones: NAL (nalidixic acid), CIP (ciprofloxacin); Tetracyclines: TET (tetracycline); Macrolides: ERY (erytromycin); Aminoglycosides: GEN (gentamycin)

^b^Number of different antimicrobial classes per resistance profile

^c^It includes the *C. coli* isolate

## Discussion

The role of livestock and a sympatric wild ruminant in the epidemiology of two relevant zoonotic agents—*Campylobacter* and *Salmonella*—in an alpine ecosystem has been assessed. It has been found a differential prevalence of these pathogens, and a distinct relevance of the host species studied.

Of the four host species included in our study, cattle were the main carrier of *Campylobacter* in alpine environments. The prevalence found in our study area was higher than that previously reported in cattle under free-ranging and extensive farming management in Spain [15, 25]. However, similar frequencies have been described in Spain as well as in other European countries for dairy cattle, either under indoor or intensive farming management [26–28]. Nevertheless, seasonal, environmental and specific management variables must be taken into account as they all can affect the carriage rate of *Campylobacter* [28]. In our study, the high burden detected can be due to the winter farming conditions. During this season, animals are gathered in the lower mountain pastures and may be kept indoor for long periods of time during colder days, increasing the chance of infection from faecal material [25].

The low prevalence detected in sheep indicates that this species has a moderate role in the epidemiology of *Campylobacter* in alpine environments. Sheep, as cattle, is usually pointed as an important source of enterobacterial contamination in the environment and the recovery rates are usually higher than in our study ([5, 29] but see [25]). Findings of enteropathogens in horses are rarely reported, particularly in free-ranging horses for meat production, but studies have shown that horses are potential carriers of *Campylobacter* [30] and have the potential to cause disease outbreaks in humans by contaminating the water supply [31]. However, in our study we did not detect any positive horse, highlighting the null or negligible role of free-ranging horses in the epidemiology of *Campylobacter* in alpine environments.

The lack of transmission from livestock to chamois may be due to a spatial behaviour adjustment of chamois to avoid pastures when livestock is present [32], or even an avoidance of dung-contaminated areas [33]. However, this spatial avoidance has not been documented between livestock species and the lack of transmission may be due to other variables. In fact, studies have shown that in faeces shed on pastures, *Campylobacter* rapidly inactivates in spring and summer due to desiccation, which reduces the risk of transmission [34].

The high genetic diversity we have found among *C. jejuni* isolates is in accordance with other reports in livestock [25], and is common in this bacterial species due to its genetic instability [35]. In situation of environmental stress, this genomic variability seems to be relevant, as it generates a population heterogeneity to improve fitness and survival in hostile environments, increasing the colonization potential [36]. On the other hand, the host-species specificity of *C. jejuni* we have found in ungulates has also been reported in other host species, particularly birds [37].

*Salmonella* spp. was not found in the livestock species from our study area. Even if *Salmonella* is principally isolated from poultry or pigs [38, 39], low to moderate prevalence are usually expected in cattle and sheep in Spain [6, 40]. In wild ruminants, the occurrence of *Salmonella* is frequently low or null [40, 41]. Also, this zoonotic agent has been seldom isolated in chamois species [42, 43], although mortality due to septicaemia has been described [44]. It has also been reported a reduction in the reproductive success of alpine chamois (*Rupicapra rupicapra*) in relation with the prevalence of antibodies against this pathogen [45]. The low prevalence in our study area seems to indicate that this pathogen is not a problem for chamois demography. In fact, the overall lack of detection of *Salmonella* in livestock suggest a very low burden in alpine environments.

In our study, cattle and sheep showed to be reservoirs of AMR bacteria in alpine environments, and therefore can contribute to their spread and maintenance in these habitats. We found resistance to antimicrobial agents in all but two *Campylobacter* isolates tested, with high frequencies of resistance to nalidixic acid, ciprofloxacin and tetracycline. These findings are similar to what has been reported for *Campylobacter* in the EU survey on AMR in zoonotic bacteria, where resistance to these agents were the most frequently detected in livestock in Europe in 2017 [18]. Results regarding tetracycline resistance are not surprising as they are the most used antimicrobials in livestock in Europe [46]. However, the frequencies of resistance to quinolones (i.e. nalidixic acid) and fluoroquinolones (i.e. ciprofloxacin) are alarming as they are higher than those previously described in Europe for livestock [18, 47, 48]. On the other hand, a very low occurrence of resistance to macrolides and aminoglycosides was found, probably due to the relatively infrequent use of these antibiotics in food-producing species [46].

Wildlife is frequently considered sentinel of the burden and distribution of pathogens and AMR in the environment [49, 50]. Due to their life history, synurbic wildlife [23], small mammals near aquatic environments [24] and even seabirds around human settlements in Antarctica [12] show a strong link between environmental contamination and the wildlife carriage of antimicrobial-resistant pathogens. However, data obtained in our study in Pyrenean chamois does not reflect the high levels of AMR enteropathogens spreading by sympatric livestock. In fact, the scarce information in alpine environments suggests that wild mammals are uncommonly infected by *Campylobacter* and *Salmonella* [51, 52], supporting that the pathogenic burden of alpine environments is low, despite the high levels of excretion by livestock. Even fewer studies have investigated the presence of AMR pathogens in those species and have reached similar conclusions [53].

The recent agricultural shift in European mountains, where primary production is gradually abandoned [54] and the replacement of sheep for cattle by the remaining farmers [55], is probably producing constant changes in the epidemiology of zoonotic enteopathogens in these areas. Locally we may predict that the presence of *Campylobacter* will increase in areas where the densities of cattle increase but may decrease in areas of farm abandonment. However, additional studies on *Campylobacter* and *Salmonella* survival in alpine environments are needed to better understand the cycles of environmental contamination and AMR spread.

## Conclusions

In conclusion, we show that free-ranging cattle and sheep are spreaders of enteric zoonotic bacteria of public health concern as well as their AMR strains in the alpine environment. Therefore, contaminated alpine pastures or streams may constitute a source for the dissemination of these organisms. However, alpine wild ungulates such as Pyrenean chamois might not be considered as potential bioindicators or sentinels of the pathogen (AMR or susceptible) burden of this environment.

## Methods

### Study area and sampling

The study was performed from 2015 to 2017 in the Freser-Setcases National Game Reserve (FSNGR), eastern Pyrenees, Catalonia, Spain (42˚ 22’ N, 2˚ 09’ E). The FSNGR is a mountainous area of 20.200 ha where subalpine and alpine ecosystems predominate with an average altitude of 2,000 meters above sea level (m.a.s.l.), ranging from 1,200 to 2,910 m.a.s.l. The area is inhabited by a population of around 3,000 Pyrenean chamois (*Rupicapra p. pyrenaica*), the most abundant wild ungulate present. Around 300 specimens are legally hunted every year.. In the FSNGR, around 4,660 cattle, 1,450 sheep and 310 horses benefit from these conditions [56].

We collected 86 faecal samples from harvested Pyrenean chamois during the hunting season. We obtained samples from the rectum of the animal using disposable gloves, minutes after being hunted. Additionally, we collected fresh faeces from livestock (up to 100 g) from the ground just after deposition. We obtained samples from 74 cattle from 19 herds, 39 sheep from 3 flocks and 30 horses from 10 herds. Special care was taken to avoid sample contamination and we used individual disposable plastic bags for every sample. All samples were refrigerated and processed within 24 h.

### Isolation and identification of *Campylobacter* spp. and *Salmonella* spp

For each sample, we introduced ten grams of faeces into sterile test tubes to which we added between three to five mL of sterile water to homogenize and facilitate its handling using a sterile swab.

#### *Campylobacter* isolation and identification

We performed *Campylobacter* isolation by direct streaking onto blood-free selective medium (mCCDA, modified charcoal cefoperazone desoxycholate agar, CM739 with selective supplement, SR0155E; Oxoid, Basingstoke, UK) as described by Urdaneta et al. [57]. Plates were incubated at 42ºC for 48 h under microaerobic conditions, and up to four presumptive colonies per positive sample were subcultured onto blood agar plates (BioMerieux, Marcy l’Etoile, France) at 37ºC during 48 h in microaerobic conditions. We identified *Campylobacter* species by multiplex PCR using primers targeting the lipid A gene lpxA [58]. All isolates were preserved in brain-hearth infusion broth with 20 % of glycerol at -75ºC for later analysis.

#### *Salmonella* isolation and identification

*Salmonella* isolation procedure was as described by Antilles et al. [10]. Briefly, we pre-enriched the swabs in buffered peptone water (Oxoid, Basingstoke, UK) at 37ºC for 20 ± 2 h, followed by a selective enrichment in Rappaport-Vassiliadis broth (Oxoid, Basingstoke, UK) at 42ºC for 24–48 h and subculturing onto XLT4 (Xylose Lysine Tergitol 4) agar plates (Merck, Darmstadt, Germany) at 37ºC for 24 h. Finally, up to four presumptive colonies were selected and streaked onto MacConkey agar and incubated at 37ºC for 24 h. We confirmed the lactose-negative colonies as *Salmonella* spp. with the Mucap (Biolife, Milano, Italy) and indole tests. *Salmonella* serotyping was carried out according to the White-Kauffmann-Le Minor scheme [59] at the *Laboratori Agroalimentari* (Cabrils, Spain) of the *Departament d’Agricultura, Ramaderia, Pesca i Alimentació*. Isolates were preserved in brain-hearth infusion broth with 20 % of glycerol at -75ºC for later analysis.

### Molecular typing of *Campylobacter* and *Salmonella* isolates

We determined the genotypic diversity among *Campylobacter* isolates by *fla*A-RFLP, following the CAMPYNET protocol as previously described by Harrington et al., [60]. The *fla*A gene was amplified using the forward A1 (5’-GGA TTT CGT ATT AAC ACA AAT GGT GC -3’) and reverse A2 (5’- CTG TAG TAA TCT TAA AAC ATT TTG-3’) primers [61]. The amplified product of 17.7 kb was digested using the restriction enzyme DdeI (*Hyp*F3I; FastDigest, Thermo Fisher Scientific, Waltham, MA, USA). The digest products were separated by electrophoresis on 2.5 % agarose gel in 1x TAE buffer at 90V for 3 h.

We analysed the RFLP band patterns using Fingerprinting II v3.0 software (Bio-Rad, Hercules, CA, USA). We calculated the similarity matrices using the Dice coefficient with tolerance and optimization values of 1.0 %. We constructed a dendrogram based on an unweighted-pair group method with arithmetic mean (UPGMA) cluster analysis. We used a cut-off of 90 % for the determination of the different profiles.

We genotyped the *Salmonella* isolates by ERIC-PCR as previously described [10], except that we used a 50ºC annealing temperature that is more adequate for Enterobacteriaceae. We used the primer pairs ERIC-F (5’-AAG TAA GTG ACT GGG GTG AGC G-3’) and ERIC-R (5’-ATG TAA GCT CCT GGG GAT TCA C-3’) [62].

### Antimicrobial susceptibility testing

We considered all *Campylobacter* isolates from the same individual showing identical *fla*A-RFLP profiles as the same strain and we only selected one of them for the antimicrobial susceptibility testing. We tested the isolates using a minimum inhibitory concentration (MIC)-based broth microdilution (EUCAMP2 Sensititre® Susceptibility plates, ThermoFisher Scientific, Spain). The antimicrobials tested were: nalidixic acid (1–64 mg/L), ciprofloxacin (0.12–16 mg/L), tetracycline (0.5–64 mg/L), gentamycin (0.12–16 mg/L), streptomycin (0.25–16 mg/L) and erythromycin (1–128 mg/ L). We used *C. jejuni* ATCC 33,560 as control strain. Plates were incubated at 37ºC for 48 h.

*Salmonella* susceptibility testing was performed using EUVSEC plates (Sensititre® Susceptibility plates, ThermoFisher Scientific, Spain). Antimicrobials tested were: ampicillin (1–64 mg/L), cefotaxime (0.25-1 mg/L), ceftazidime (0.5–8 mg/L), meropenem (0.03–16 mg/L), nalidixic acid (4–128 mg/L), ciprofloxacin (0.015–8 mg/L), tetracycline (2–64 mg/L), tigecycline (0,25–8 mg/L), gentamycin (0.5–32 mg/L), azithromycin (2–64 mg/L), chloramphenicol (8–128 mg/L), trimethoprim (0,25–32 mg/L), sulfamethoxazole (8–1,024 mg/L), and colistin (1–16 mg/L). We used *E. coli* ATCC 25,922 as control strain. Plates were incubated at 37ºC for 24 h.

An isolate was considered MDR when showing resistance to three or more nonrelated antimicrobials. We used epidemiological cut-off values according to EUCAST guidelines (www.eucast.org) to consider an isolate susceptible or resistant. When reporting data using EUCAST epidemiological cut-off values, bacteria should be reported as ‘‘wild-type’’ (WT) or ‘‘non-wild-type’’ (non-WT) [63]. For simplicity, the terms “susceptible” and “resistant” have been used here.

### Data analyses

The prevalence of each pathogen was calculated from the proportion of positives to the total number of animals examined, with Wilson score confidence intervals of 95 %.

## Data Availability

The datasets used and/or analysed during the current study are available from the corresponding author on reasonable request.

## Abbreviations

AMR: Antimicrobial resistance
EUCAST: European Committee on Antimicrobial Susceptibility Testing
flaA-RFLP: Restriction fragment length polymorphism of the *fla*A gene
MDR: Multidrug resistant
WT: Wild-type

## References

[1] Havelaar AH, Kirk MD, Torgerson PR, Gibb HJ, Hald T, Lake RJ, et al. World Health Organization global estimates and regional comparisons of the burden of foodborne disease in 2010. PLOS Med. 2015;12(12):e1001923. von Seidlein L, editor 10.1371/journal.pmed.1001923 PMC4668832 26633896

[2] EFSA and ECDC (European Food Safety Authority and European Centre for Disease Prevention and Control). The European Union summary report on trends and sources of zoonoses, zoonotic agents and food-borne outbreaks in 2017. EFSA J 2018. 2018;16(12). 10.2903/j.efsa.2018.5500 PMC7009540 32625785

[3] Pires SM, Vigre H, Makela P, Hald T. Using outbreak data for source attribution of human salmonellosis and campylobacteriosis in Europe. Foodborne Pathog Dis. 2010 Nov;7(11):1351–61. 10.1089/fpd.2010.0564 20586609

[4] Taylor EV, Herman KM, Ailes EC, Fitzgerald C, Yoder JS, Mahon BE (2013). Common source outbreaks of *Campylobacter* infection in the USA, 1997–2008. Epidemiol Infect.

[5] Stanley K, Jones K (2003). Cattle and sheep farms as reservoirs of Campylobacter. J Appl Microbiol..

[6] Hurtado A, Ocejo M, Oporto B. *Salmonella* spp. and *Listeria monocytogenes* shedding in domestic ruminants and characterization of potentially pathogenic strains. Vet Microbiol. 2017 Oct;210(September):71–6. 10.1016/j.vetmic.2017.09.003 29103699

[7] Dorner SM, Huck PM, Slawson RM. Estimating potential environmental loadings of *Cryptosporidium* spp. and *Campylobacter* spp. from livestock in the Grand River Watershed, Ontario, Canada. Environ Sci Technol. 2004;38(12):3370–80. 10.1021/es035208+ 15260337

[8] Carbonero A, Paniagua J, Torralbo A, Arenas-Montes A, Borge C, García-Bocanegra I (2014). *Campylobacter* infection in wild artiodactyl species from southern Spain: Occurrence, risk factors and antimicrobial susceptibility. Comp Immunol Microbiol Infect Dis.

[9] Hilbert F, Smulders FJM, Chopra-Dewasthaly R, Paulsen P (2012). *Salmonella* in the wildlife-human interface. Food Res Int.

[10] Antilles N, Sanglas A, Cerdà-Cuéllar M (2015). Free-living waterfowl as a source of zoonotic bacteria in a dense wild bird population area in northeastern Spain. Transbound Emerg Dis.

[11] Moré E, Ayats T, Ryan PG, Naicker PR, Keddy KH, Gaglio D, et al. Seabirds (Laridae) as a source of *Campylobacter* spp., *Salmonella* spp. and antimicrobial resistance in South Africa. Environ Microbiol. 2017;19(10):4164–76. 10.1111/1462-2920.13874 28752962

[12] Cerdà-Cuéllar M, Moré E, Ayats T, Aguilera M, Muñoz-González S, Antilles N (2019). Do humans spread zoonotic enteric bacteria in Antarctica?. Sci Total Environ..

[13] Hassell JM, Ward MJ, Muloi D, Bettridge JM, Robinson TP, Kariuki S (2019). Clinically relevant antimicrobial resistance at the wildlife–livestock–human interface in Nairobi: an epidemiological study. Lancet Planet Heal.

[14] Sippy R, Sandoval-Green CMJ, Sahin O, Plummer P, Fairbanks WS, Zhang Q (2012). Occurrence and molecular analysis of *Campylobacter* in wildlife on livestock farms. Vet Microbiol.

[15] Navarro-Gonzalez N, Ugarte-Ruiz M, Porrero MC, Zamora L, Mentaberre G, Serrano E (2014). *Campylobacter* Shared Between Free-Ranging Cattle and Sympatric Wild Ungulates in a Natural Environment (NE Spain). Ecohealth.

[16] Jasovský D, Littmann J, Zorzet A, Cars O (2016). Antimicrobial resistance—a threat to the world’s sustainable development. Ups J Med Sci..

[17] Bengtsson-Palme J, Kristiansson E, Larsson DGJ (2018). Environmental factors influencing the development and spread of antibiotic resistance. FEMS Microbiol Rev.

[18] EFSA (European Food Safety Authority) and ECDC (European Centre for Disease Prevention and Control). The European Union summary report on antimicrobial resistance in zoonotic and indicator bacteria from humans, animals and food in 2017. EFSA J 2019. 2019;17(2). 10.2903/j.efsa.2019.5598 PMC7009238 32626224

[19] Smith DL, Dushoff J, Morris JG (2005). Agricultural antibiotics and human health. PLoS Med.

[20] Silbergeld EK, Graham J, Price LB (2008). Industrial food animal production, antimicrobial resistance, and human health. Annu Rev Public Health..

[21] Ramey AM, Ahlstrom CA (2020). Antibiotic resistant bacteria in wildlife: perspectives on trends, acquisition and dissemination, data gaps, and future directions. J Wildl Dis..

[22] Mercat M, Clermont O, Massot M, Ruppe E, De Garine-Wichatitsky M, Miguel E (2016). *Escherichia coli* population structure and antibiotic resistance at a buffalo/cattle interface in southern Africa. Appl Environ Microbiol.

[23] Jobbins SE, Alexander KA (2015). From whence they came—Antibiotic-resistant *Escherichia coli* in African wildlife. J Wildl Dis.

[24] Furness LE, Campbell A, Zhang L, Gaze WH, McDonald RA (2017). Wild small mammals as sentinels for the environmental transmission of antimicrobial resistance. Environ Res.

[25] Oporto B, Esteban JI, Aduriz G, Juste RA, Hurtado A (2007). Prevalence and strain diversity of thermophilic campylobacters in cattle, sheep and swine farms. J Appl Microbiol.

[26] Vilar M, Peña F, Pérez I, Diéguez F, Sanjuán M, Rodríguez-Otero J (2010). Presence of *Listeria*, *Arcobacter*, and *Campylobacter* spp. in dairy farms in Spain. Berl Munch Tierarztl Wochenschr.

[27] Nielsen EM (2002). Occurrence and strain diversity of thermophilic campylobacters in cattle of different age groups in dairy herds. Lett Appl Microbiol.

[28] Ellis-Iversen J, Cook AJC, Smith RP, Pritchard GC, Nielen M (2009). Temporal Patterns and Risk Factors for Escherichia coli O157 and Campylobacter spp&nbsp;Young Cattle. J Food Prot..

[29] Uaboi-Egbenni PO, Bessong PO, Samie A, Obi CL (2010). Campylobacteriosis in sheep in farm settlements in the Vhembe District of South Africa. African J Microbiol Res.

[30] Moriarty E, Downing M, Bellamy J, Gilpin B (2015). Concentrations of faecal coliforms, *Escherichia coli*, enterococci and *Campylobacter* spp. in equine faeces. N Z Vet J.

[31] Paruch L, Paruch AM, Sørheim R (2020). DNA-based faecal source tracking of contaminated drinking water causing a large *Campylobacter* outbreak in Norway 2019. Int J Hyg Environ Health.

[32] Chirichella R, Ciuti S, Apollonio M (2013). Effects of livestock and non-native mouflon on use of high-elevation pastures by Alpine chamois. Mamm Biol - Zeitschrift für Säugetierkd..

[33] Fankhauser R, Galeffi C, Suter W (2008). Dung avoidance as a possible mechanism in competition between wild and domestic ungulates: Two experiments with chamois *Rupicapra rupicapra*. Eur J Wildl Res.

[34] Moriarty EM, Mackenzie ML, Karki N, Sinton LW. Survival of *Escherichia coli*, Enterococci, and *Campylobacter* spp. in Sheep Feces on Pastures. Appl Environ Microbiol. 2011;77(5):1797–803. 10.1128/AEM.01329-10 PMC3067260 21239546

[35] de Boer P, Wagenaar JA, Achterberg RP, van Putten JPM, Schouls LM, Duim B (2002). Generation of *Campylobacter jejuni* genetic diversity in vivo. Mol Microbiol.

[36] Ridley AM, Toszeghy MJ, Cawthraw SA, Wassenaar TM, Newell DG (2008). Genetic instability is associated with changes in the colonization potential of Campylobacter jejuni in the avian intestine. J Appl Microbiol..

[37] Griekspoor P, Colles FM, McCarthy ND, Hansbro PM, Ashhurst-Smith C, Olsen B (2013). Marked host specificity and lack of phylogeographic population structure of *Campylobacter jejuni* in wild birds. Mol Ecol.

[38] Davies RH, Dalziel R, Gibbens JC, Wilesmith JW, Ryan JMB, Evans SJ (2004). National survey for *Salmonella* in pigs, cattle and sheep at slaughter in Great Britain (1999–2000). J Appl Microbiol.

[39] Solveig J, Trude ML, Merete H, Bjarne B, Torkjel B, Michaela F, et al. The surveillance and control programme for *Salmonella* in live animals, eggs and meat in Norway. Annual Report. Oslo; 2008.

[40] Navarro-Gonzalez N, Velarde R, Porrero MC, Mentaberre G, Serrano E, Mateos A (2014). Lack of Evidence of Spill-Over of *Salmonella* enterica Between Cattle and Sympatric Iberian ibex (*Capra pyrenaica*) from a Protected Area in Catalonia, NE Spain. Transbound Emerg Dis.

[41] Díaz-Sánchez S, Sánchez S, Herrera-León S, Porrero C, Blanco J, Dahbi G, et al. Prevalence of Shiga toxin-producing *Escherichia coli*, *Salmonella* spp. and *Campylobacter* spp. in large game animals intended for consumption: Relationship with management practices and livestock influence. Vet Microbiol. 2013;163(3–4):274–81. 10.1016/j.vetmic.2012.12.026 23384892

[42] Obwegeser T, Stephan R, Hofer E, Zweifel C (2012). Shedding of foodborne pathogens and microbial carcass contamination of hunted wild ruminants. Vet Microbiol.

[43] Dumitrescu V, Borlea F, Nichita I, Bucur IM, Tîrziu E. Comparative research on antimicrobial resistance in bacteria isolated from domestic and wild animals (chamois - *Rupicapra rupicapra*). In: Young People and Veterinary Medicine Research. Timișoara, Romania; 2018. p. 53.

[44] Glawischnig W, Khaschabi D, Schöpf K, Schönbauer M (2000). An outbreak of *Salmonella* Dublin in chamois (Rupicapra rupicapra). Wien Tierarztl Monatsschr.

[45] Pioz M, Loison A, Gibert P, Jullien J-M, Artois M, Gilot-Fromont E (2008). Antibodies against *Salmonella* is associated with reduced reproductive success in female alpine chamois (Rupicapra rupicapra). Can J Zool.

[46] EMA (European Medicines Agency). Sales of veterinary antimicrobial agents in 31 European countries in 2017. Trends from 2010 to 2017 (EMA/294674/2019). Amsterdam; 2019.

[47] Ocejo M, Oporto B, Hurtado A (2019). Occurrence of Campylobacter jejuni and Campylobacter coli in cattle and sheep in northern Spain and changes in antimicrobial resistance in two studies 10-years apart. Pathogens..

[48] Sproston EL, Wimalarathna HML, Sheppard SK. Trends in fluoroquinolone resistance in *Campylobacter*. Microb Genomics. 2018;4(8). 10.1099/mgen.0.000198 PMC6159550 30024366

[49] White A, Hughes JM (2019). Critical Importance of a One Health Approach to Antimicrobial Resistance. Ecohealth..

[50] Conrad PA, Meek LA, Dumit J (2013). Operationalizing a One Health approach to global health challenges. Comp Immunol Microbiol Infect Dis.

[51] Marreros N, Hüssy DH, Albini S, Frey CF, Abril C, Vogt HR (2011). Epizootiologic investigations of selected abortive agents in free-ranging alpine ibex (*Capra ibex ibex*) in Switzerland. J Wildl Dis.

[52] Pagano A, Nardi G, Bonaccorso C, Falbo V, Passi C, Sanguinetti V (1985). Faecal bacteria of wild ruminants and the alpine marmot. Vet Res Commun.

[53] Caprioli A, Donelli G, Falbo V, Passi C, Pagano A, Mantovani A (1991). Antimicrobial resistance and production of toxins in *Escherichia coli* strains from wild ruminants and the alpine marmot. J Wildl Dis.

[54] MacDonald D, Crabtree J, Wiesinger G, Dax T, Stamou N, Fleury P (2000). Agricultural abandonment in mountain areas of Europe: Environmental consequences and policy response. J Environ Manage.

[55] Garcia-Ruiz JM, Lasanta-Martinez T (1990). Land-Use Changes in the Spanish Pyrenees. Mt Res Dev.

[56] Idescat (2009). Institut d’Estadística de Catalunya).

[57] Urdaneta S, Dolz R, Cerdà-Cuéllar M (2015). Assessment of two different types of sample for the early detection and isolation of thermophilic *Campylobacter* in broiler farms. Avian Pathol.

[58] Klena JD, Parker CT, Knibb K, Ibbitt JC, Devane PML, Horn ST (2004). Differentiation of Campylobacter coli, Campylobacter jejuni, Campylobacter lari, and Campylobacter upsaliensis by a multiplex PCR developed from the nucleotide sequence of the lipid A gene lpxA. J Clin Microbiol.

[59] Grimont P, Weill F-X. Antigenic formulae of the Salmonella servovars. 9th Edition. WHO Collaborating Centre for reference and research on *Salmonella* (WHOCC-SALM). Paris:; 2007.

[60] Harrington CS, Moran L, Ridley AM, Newell DG, Madden RH (2003). Inter-laboratory evaluation of three flagellin PCR/RFLP methods for typing *Campylobacter jejuni* and *C. coli*: the CAMPYNET experience. J Appl Microbiol.

[61] Nachamkin I, Bohachick K, Patton CM (1993). Flagellin gene typing of *Campylobacter jejuni* by restriction fragment length polymorphism analysis. J Clin Microbiol.

[62] Versalovic J, Koeuth T, Lupski JR (1991). Distribution of repetitive DNA sequences in eubacteria and application to fingerprinting of bacterial genomes. Nucleic Acids Res.

[63] Schwarz S, Silley P, Simjee S, Woodford N, van Duijkeren E, Johnson AP (2010). Editorial: Assessing the antimicrobial susceptibility of bacteria obtained from animals. J Antimicrob Chemother..

